# TikTok as a Source of Information on Temporomandibular Disorders: Reliable Health Education or Misinformation?

**DOI:** 10.4317/jced.62754

**Published:** 2025-10-17

**Authors:** Maria Amália Dias Pereira Calças, Caio Sberni Pinheiro de Souza, Samira Guimarães Andrade, Luiz Guilherme Spadon-Brito, Alex Moreira Mélo, Melissa de Oliveira Melchior, Jardel Francisco Mazzi-Chaves, Laís Valencise Magri

**Affiliations:** 1 DDS. Department of Restorative Dentistry, School of Dentistry of Ribeirão Preto, University of São Paulo (USP), Ribeirão Preto, SP, Brazil; 2 DDS. MSc. Department of Restorative Dentistry, School of Dentistry of Ribeirão Preto, University of São Paulo (USP), Ribeirão Preto, SP, Brazil; 3 DDS, MSc, PhD, and remain correctly listed under the Department of Restorative Dentistry, School of Dentistry of Ribeirão Preto, University of São Paulo (USP), Ribeirão Preto, SP, Brazil

## Abstract

**Background:**

Temporomandibular disorders (TMD) are prevalent musculoskeletal conditions affecting the orofacial region, often requiring interdisciplinary management. Social media platforms, particularly TikTok, have become popular sources of health-related information. However, concerns persist regarding the reliability and educational value of user-generated content. Objective: This study aimed to evaluate the reliability and educational value of TikTok videos on TMD and compare content characteristics across three categories of creators: general users, dental care professionals, and other healthcare professionals.

**Material and Methods:**

A descriptive observational cross-sectional study was conducted, analyzing TikTok videos related to TMD using predefined inclusion criteria. A total of 98 videos were assessed based on three validated scoring systems: Video Content Score (VCS), Quality Criteria for Consumer Health Information (DISCERN), and Global Quality Score (GQS). Inter-rater reliability was established (Cohen's kappa 0.86). Statistical analyses, including the Kruskal-Walli's test and post-hoc Dunn's test, were performed to compare video characteristics and engagement metrics among content creator groups. It was also performed a quali-quantitative analysis of the scoring systems.

**Results:**

The analyzed TikTok videos on TMD showed low educational quality (VCS = 2.0, GQS = 2.0, median) and poor to fair reliability (DISCERN = 33.0), highlighting their limited and incomplete information. Although no statistically significant differences were found among content creator groups (VCS: p = 0.453; DISCERN: p = 0.239; GQS: p = 0.341), videos by healthcare professionals tended to have higher quality scores, presenting more structured content aligned with scientific guidelines on TMD. The analyzed TikTok videos on TMD exhibit critical deficiencies, including inadequate assessment, classification, and discussion of etiology and risk factors. Low VCS, DISCERN, and GQS scores highlight the lack of evidence-based content, poor reliability, and limited educational value, reinforcing concerns about misinformation

**Conclusions:**

Despite TikTok's growing role in disseminating health-related information, the overall educational quality of TMD-related videos remains inadequate. While healthcare professionals tend to produce more structured content, the lack of significant differences across creator groups underscores a general deficiency in reliable, evidence-based information on the platform. These findings highlight the need for greater oversight, content validation, and expert-reviewed educational initiatives to improve the credibility of TMD-related information on social media.

## Introduction

Temporomandibular disorders (TMD) encompass a set of musculoskeletal conditions affecting the temporomandibular joints, masticatory muscles, and adjacent structures (1). The most common symptoms associated with TMD include orofacial pain, restricted mandibular movement, and joint noises such as clicking and crepitus (2 , 3). TMD represents the primary cause of non-dental orofacial pain, and its diagnosis is often complex due to its multifactorial etiology, which involves biopsychosocial factors. Additionally, the similarity of its symptoms to other chronic pain conditions, such as tension-type headaches and fibromyalgia, further complicates diagnosis (4 - 6).

Although dental caries and periodontal disease remain the most prevalent oral health problems, the demand for TMD-related care has increased significantly. Epidemiological studies indicate that TMD affects approximately 5% to 12% of the general population (7). Furthermore, Valesan et al. report that approximately 31% of adults and older individuals experience TMD symptoms at some point in their lives, while the prevalence among children and adolescents is around 11% (6). Global prevalence rates also vary, with TMD affecting 33% of the population in Asia, 47% in South America, 26% in North America, and 29% in Europe (8). These figures highlight the significant impact of TMD on public health (2).

Given this complexity, a multidisciplinary approach involving dentists, psychologists, and physiotherapists, among other healthcare professionals, is necessary to ensure accurate diagnosis and appropriate treatment planning (3 - 5). TMD symptoms can impair essential oral functions, causing pain and discomfort severe enough to interfere with chewing and speaking, ultimately affecting daily activities, social interactions, and psychological well-being. The condition has a substantial impact on quality of life and overall well-being, prompting an increasing number of individuals to seek healthcare services for diagnosis and management (9 - 11).

Concurrently, social media platforms have evolved beyond mere entertainment to become valuable tools for disseminating health-related information (12 , 13). Platforms such as TikTok, YouTube, Facebook, Instagram, and X provide users with easy access to health-related content and enable the sharing of personal experiences (12 , 14). Currently, many individuals prefer searching for health information on social media rather than consulting healthcare professionals, which raises concerns about the accuracy and reliability of the information accessed (15 , 16).

Among these platforms, TikTok has emerged as a dominant video-sharing application, being the most downloaded social media app in 2020 and now boasting over one billion users with billions of daily views. The average user spends approximately 52 minutes per day on the platform (16 , 17). Recently, TikTok has gained prominence in the medical and dental fields as an effective tool for obtaining and disseminating public health information. The platform provides a wide range of short, freely accessible videos with educational value, benefiting both patients and healthcare professionals (14 , 18 , 19).

Social media has transformed health education by offering opportunities for academic engagement, networking, and professional development. Additionally, these platforms have reshaped patient awareness regarding key public health issues, aiding in diagnosis and treatment (17 , 20). Concurrently, the open science movement advocates for increased visibility, productivity, and circulation of scientific knowledge through the promotion of a sharing culture, aiming to socialize knowledge and facilitate the redistribution, reuse, and reproducibility of research data (21 , 22). Open science fosters transparency and accessibility in research, which is essential for improving the quality of information regarding TMD (23). Collaboration between healthcare professionals and content creators, fueled by open science, can ensure that information disseminated on platforms such as TikTok is both accurate and accessible, thus combating misinformation (23 , 24).

However, the educational value of this information is a matter of concern, as although social media platforms such as TikTok have the potential to disseminate health-related concepts in an accessible and engaging manner, the accuracy and reliability of such information are often questionable. This is mainly due to the lack of auditability, which arises from the vast amount of content and users, who are sometimes not healthcare professionals qualified for the topic (15 , 16 , 20 , 25). Given the high prevalence of TMD and the vast number of TikTok users seeking and sharing information on the platform, TMD-related content is widely disseminated. However, research evaluating the educational value and reliability of this content remains scarce.

The problematic dissemination of health information on social media has reshaped how the public accesses and interprets scientific content yet concerns persist regarding the reliability and accuracy of such information. In the context of TMD, a prevalent condition with significant implications for quality of life, the widespread use of TikTok as a health information source raises concerns about the spread of misleading or incomplete content. The relevance of this study lies in assessing the extent to which TikTok videos contribute to public knowledge about TMD, considering that unregulated user-generated content may influence clinical decision-making, reinforce misconceptions, and lead to inadequate self-diagnosis or treatment approaches. Given the increasing preference for social media over professional consultations, evaluating the scientific, social, and educational impact of such content is crucial. This study aims to address this gap by systematically analyzing the educational value and reliability of TikTok videos related to TMD and comparing content quality across different creator categories. The hypothesis is that videos produced by healthcare professionals-particularly dental professionals-demonstrate higher scientific rigor and educational quality than those created by lay users. Additionally, it is expected that while user engagement may be higher for popular content, the reliability of these videos will be limited, highlighting the necessity of expert-driven initiatives to improve the dissemination of evidence-based health information on social media platforms. Therefore, this study aims to analyze the reliability and educational value of videos available on TikTok as a source of information on TMD. Additionally, the study compared the video characteristics and educational value among three categories of content creators: general users, dental care professionals, and other healthcare professionals.

## Material and Methods

New TikTok accounts in both English and Portuguese were created for each search, and the computer's browsing history and cookies were cleared to ensure that prior web searches did not influence the results. This approach was implemented to enhance the reliability and validity of the findings by minimizing potential biases arising from previous interactions or search history.

- Study Design

This is a descriptive observational cross-sectional study that analyzes content shared on the social media platform TikTok to assess the availability of information related to TMD. The study focuses on videos posted by various content creators, including dental professionals, to examine the extent to which information aligns with scientifically accurate knowledge. The aim is to evaluate how dental professionals, as well as other creators, contribute to the dissemination of information about TMD, highlighting both accurate and potentially misleading content available to the public.

- Ethical Considerations

This project was exempt from submission to the Research Ethics Committee, as the data were collected from publicly available TikTok profiles. According to the guidelines set forth by the National Research Ethics Commission (CONEP), ethical review was not required for the study.

- Search Strategy

TikTok (available at www.tiktok.com) was used as the primary search platform from October to December 2024. The following hashtags were selected for the search: #DTM, #TMJD, #disfuncaotemporomandibular, and #temporomandibulardisorder, with the "all regions" setting applied. For each hashtag, the first 100 videos were selected based on their appearance order in the search results. The search was conducted using filters that included videos shared without time restrictions and ranked based on relevance (26 - 29). Subsequently, these videos were filtered according to inclusion and exclusion criteria, resulting in a sample of 98 videos for evaluation.

- Inclusion and Exclusion Criteria

The inclusion criteria for the study were as follows: only public TikTok profiles were considered, ensuring that the data collected were accessible to the public. The location setting was configured to "all regions" to capture a diverse range of content across various geographical areas. The main content of the video had to be related to TMD, ensuring the relevance of the videos to the study's focus. Additionally, only videos in Portuguese and English were included to maintain linguistic consistency and ensure comprehensibility for analysis.

The exclusion criteria were applied to ensure the quality and relevance of the data. Private profiles, accounts with fewer than 500 followers, and those with fewer than 25 posts were excluded to avoid content from less established or less active users. Duplicate videos were also removed to prevent redundancy in the dataset. Videos without audio or written explanations were excluded, as the presence of both is essential for meaningful analysis. Finally, commercial content and advertisements were excluded, as the study focused on educational and informational videos rather than promotional material.

Two investigators (M.A.D.P.C. and L.V.M.) independently screened the eligible videos based on the criteria outlined above.

- Data Collection

The investigators were calibrated in data interpretation by independently reviewing 10 randomly selected videos. Following this calibration process, the two investigators independently extracted and collected data for each included video. The data collected consisted of the following:

Uploader Information: This included the uploader's ID, the number of followers, and the type of uploader. The uploader type was categorized into three groups: (1) General users (laypersons), (2) Dental care professionals (dentists and oral health technicians), and (3) Other healthcare professionals (nurses, doctors, physiotherapists).

Video Characteristics: The characteristics recorded for each video included the video link, upload date, duration in seconds, elapsed time since upload, number of views, number of likes, number of shares, and number of comments.

Additionally, the following indices were calculated:

Viewing Index: To evaluate the popularity of the video, the view index was calculated as the number of views divided by the number of days since the video was uploaded. This metric provided insight into how frequently the video was viewed relative to the time since its posting.

Engagement Index: To assess the level of audience engagement with the video, the engagement index was calculated by summing the number of likes, comments, and shares. This index helped gauge how actively the audience interacted with the content.

- Evaluation and Scoring System

All videos were independently assessed by two reviewers, both with expertise in TMD and orofacial pain. Based on previous studies, the following scoring systems were applied to evaluate the videos (26 , 20).

Video Content Score (VCS), which evaluates the clarity, accuracy, relevance, and comprehensiveness of the video content, along with its structure, source credibility, and production quality.

Quality Criteria for Consumer Health Information (DISCERN), which assesses the reliability and quality of health information.

Global Quality Score (GQS), which evaluates the educational value of the video.

The evaluation process was carried out after both reviewers were trained to assess the videos consistently, using a preliminary evaluation of 10 randomly selected videos. This training ensured that both reviewers were aligned in their assessment approach. To measure inter-rater reliability for the DISCERN and GQS scores, the Cohen's kappa () coefficient was employed, with a minimum threshold of 0.81, indicating substantial agreement (30). Any discrepancies between the two reviewers were resolved by consulting a third author, who is also an expert in TMD and orofacial pain (M.O.M). This process ensured the accuracy and consistency of the video evaluations, thereby enhancing the reliability of the study's findings.

- Video Content Score (VCS)

The content of the videos was evaluated, and a total content score ranging from 1 to 7 was assigned based on the following topics covered in the videos:

Definition of TMD;

Definition of orofacial pain;

Classification of TMD;

Etiology and risk factors;

Impact of TMD on quality of life;

TMD assessment;

Therapeutic management of TMD.

The identification of the most relevant topics to be addressed in the videos was based on previous studies conducted on social media (26 , 31), as well as on themes frequently discussed by experts in the field of TMD.

- Quality Criteria for Consumer Health Information (DISCERN)

The overall quality of the video and the reliability of the source were assessed using the DISCERN criteria for consumer health information, a 16-item tool developed by Charnock et al. (10). Items 1 to 8 pertain to the reliability of the publication, items 9 to 15 assess the quality of the information provided, and item 16 reflects the overall quality as a source of information. Each item is scored from 1 ("no") to 5 ("yes").

DISCERN Items:

1. Are the objectives clear?

2. Does it achieve its objectives?

3. Is it relevant?

4. Is it clear what sources of information were used to compile the publication (besides the author or producer)?

5. Is it clear when the information used or reported in the publication was produced?

6. Is it balanced and impartial?

7. Does it provide details of additional sources to support the information?

8. Does it refer to areas of uncertainty?

9. Does it describe how each treatment works?

10. Does it describe the benefits of each treatment?

11. Does it describe the risks of each treatment?

12. Does it describe what would happen if no treatment were used?

13. Does it describe how treatment choices affect overall quality of life?

14. Is it clear that more than one treatment choice may be possible?

15. Does it provide support for shared decision-making?

16. Based on the responses to all the above questions, assess the overall quality of the publication as a source of information on treatment choices.

Based on the DISCERN score, the quality of the video and the reliability of the source were categorized as follows:

'Excellent' for scores of 63 or more;

'Good' for scores between 51 and 62;

'Fair' for scores between 39 and 50;

'Poor' for scores between 27 and 38;

'Very Poor' for scores below 26 points.

- Global Quality Score (GQS)

Score 1: Poor quality (DISCERN score &lt; 26), poor site flow, most information absent, highly unlikely to be useful for patients.

Score 2: Poor quality (DISCERN score between 27 and 38), poor site flow, some information present, but many important topics missing, very limited use for patients.

Score 3: Moderate quality (DISCERN score between 39 and 50), average flow, some important information adequately discussed, but other topics poorly covered, somewhat useful for patients.

Score 4: Good quality (DISCERN score between 51 and 62), good flow, most important topics covered, useful for patients.

Score 5: Excellent quality (DISCERN score of 61 or more), excellent flow, highly useful for patients.

Subsequently, videos with a score &lt; 3 were considered of low educational value, while those with a score &gt; 3 were considered of good educational value.

- Statistical analyses

Statistical analyses were performed to compare the video characteristics among the three categories of content creators: general users, dental care professionals, and other health care professionals. Descriptive statistics were calculated, with the median and interquartile range (IQR) used for each variable. As the data did not meet the assumptions of normality required for parametric tests, the Kruskal-Wallis test was employed to assess differences among the groups. A significance level of 5% (p &lt; 0.05) was considered for all tests. When significant differences were observed, post-hoc Dunn's test with Bonferroni correction was conducted to identify specific group differences. To evaluate the inter-rater reliability of the scoring process, Cohen's weighted kappa () was calculated for the DISCERN and GQS scores, with a threshold of 0.81 considered indicative of substantial agreement. It was also performed a quali-quantitative analysis of the scoring systems. All statistical analyses were conducted using JASP (version 0.18.2).

## Results

**Figure 1:**
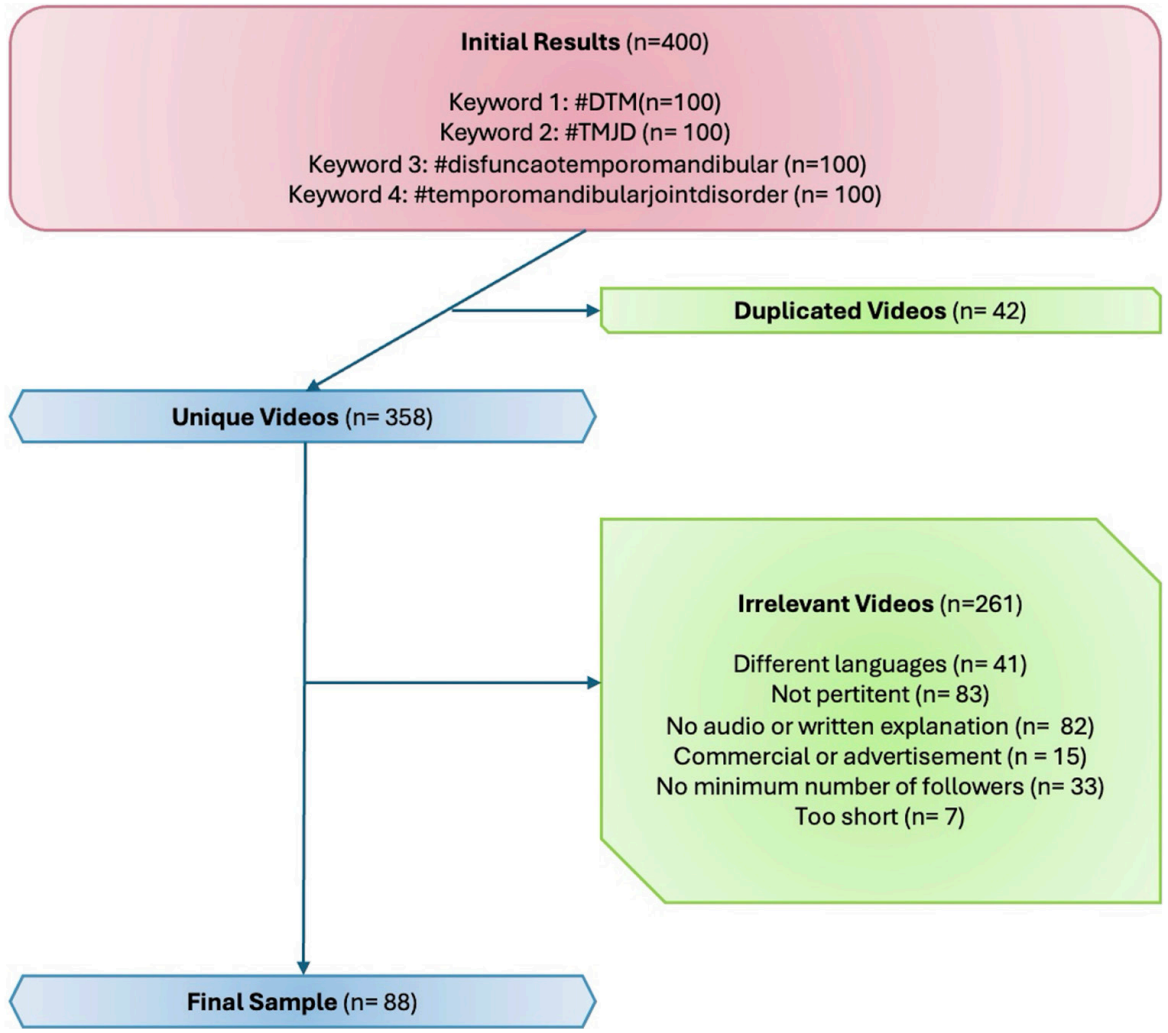
Flowchart illustrating the video selection process, resulting in 98 final videos: 4 (4.1%) from general users, 57 (60.2%) from dental care professionals, and 37 (35.7%) from other health care professionals.

Figure 1 is a flowchart illustrating the process of video selection for analysis.1 Screenshot

A total of 400 videos were initially identified using four keywords related to temporomandibular disorders (TMD). After removing 42 duplicate videos, 358 unique videos were retained. Among these, 261 videos were excluded for various reasons, such as being in different languages (41), not pertinent to the topic (73), lacking audio or written explanations (82), being commercial or advertisements (15), having fewer than the required number of followers (33), or being too short (7). The final sample for analysis consisted of 98 videos, among them, 4 videos (4.1%) were published by general users, 57 videos (60.2%) were posted by dental care professionals, and 37 videos (35.7%) were shared by other health care professionals. Inter-rater reliability analysis for the first 10 videos was performed using Cohen's kappa coefficient for VCS, DISCERN, and GQS. A threshold of 0.81 was considered adequate for agreement. The results demonstrated a kappa value of 0.86 for VCS, indicating near-perfect agreement. DISCERN and GQS both achieved a kappa value of 1.0, reflecting perfect agreement between the two raters.

A comparative analysis of video characteristics among the three categories of content creators (general users, dental care professionals, and other health care professionals) was conducted using the Kruskal-Walli's test. The results showed no statistically significant differences in most variables, including the number of followers (p = 0.131), video duration (p = 0.191), upload time (p = 0.087), and number of views (p = 0.110). However, a significant difference was observed in the number of likes (p = 0.006). A post-hoc Dunn test revealed that this difference was specifically between dental care professionals and other health care professionals (p = 0.001), indicating that videos produced by other health care professionals tend to receive more likes. These findings suggest that, while overall engagement metrics are similar across creator categories, content from other health care professionals may be more appealing or engaging to TikTok users in terms of audience interactions, (Table 1).Table 1

The analysis of the VCS, DISCERN, and GQS scores revealed relatively low median values, indicating that the educational quality and reliability of TikTok videos on TMD are inadequate. The median VCS was 2.00 (IQR = 2.00), suggesting that most videos provided limited and incomplete information about TMD. The median DISCERN score was 33.00 (IQR = 7.50), classifying the overall reliability of the videos as "poor" to "fair," according to established DISCERN thresholds. Similarly, the median GQS was 2.00 (IQR = 1.00), indicating that the educational value of these videos was generally low, falling within the "poor" to "moderate" range.

**Figure 2:**
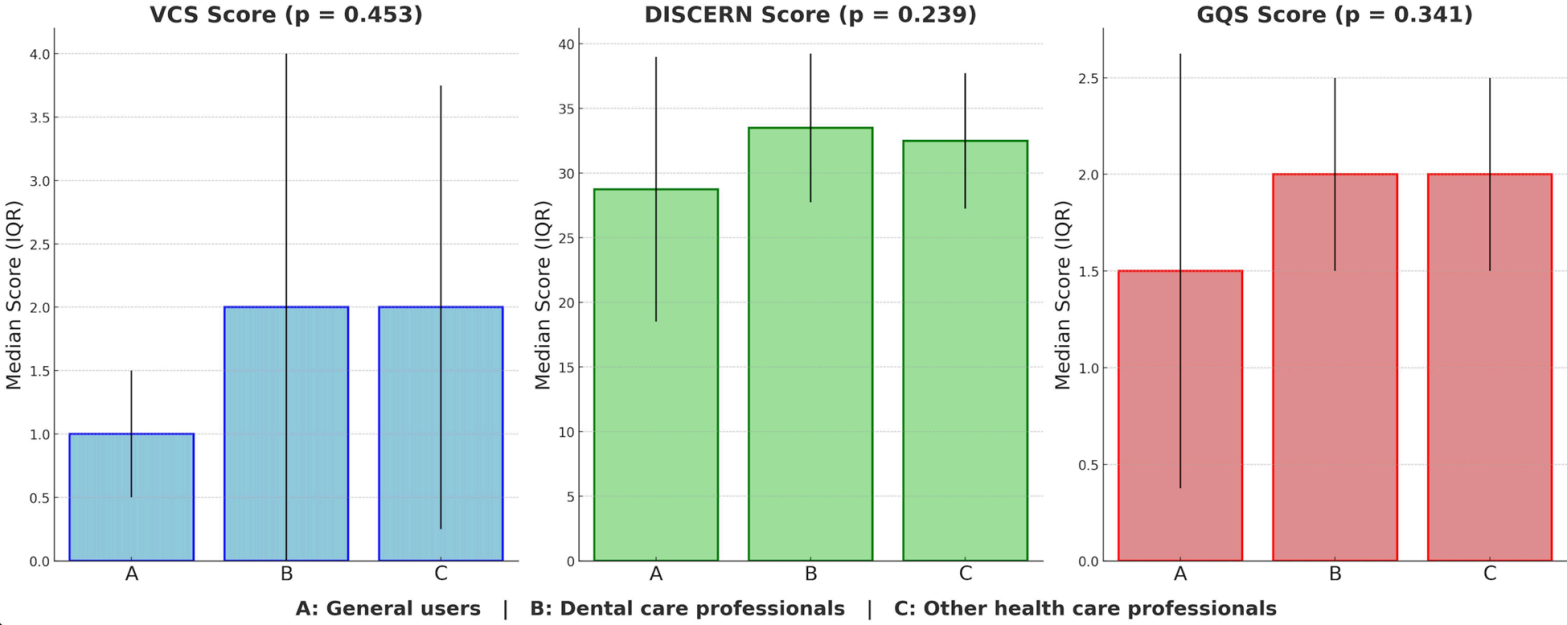
Comparison of median scores (IQR) for Video Content Score (VCS), Quality Criteria for Consumer Health Information (DISCERN), and Global Quality Score (GQS) among different categories of content creators on TikTok. Categories: A – General users, B – Dental care professionals, C – Other health care professionals. Although no statistically significant differences were found (Kruskal-Walli’s test: VCS, p = 0.453; DISCERN, p = 0.239; GQS, p = 0.341), videos produced by health professionals tended to have higher scores across all criteria.

The comparative analysis of VCS, DISCERN, and GQS scores among the three categories of content creators (general users, dental care professionals, and other health care professionals) revealed no statistically significant differences, as indicated by the Kruskal-Walli's test (VCS: p = 0.453, DISCERN: p = 0.239, GQS: p = 0.341) (Fig. 2).2 Screenshot

Despite the lack of statistical significance, a clear trend was observed, with videos produced by dental care professionals and other health care professionals consistently achieving higher median scores across all evaluated criteria. This suggests that, while not reaching statistical significance, healthcare professionals may provide more structured and reliable educational content on TMD compared to general users. These findings highlight the importance of considering both statistical and clinical relevance when evaluating the quality of health-related content on social media platforms.

**Figure 3:**
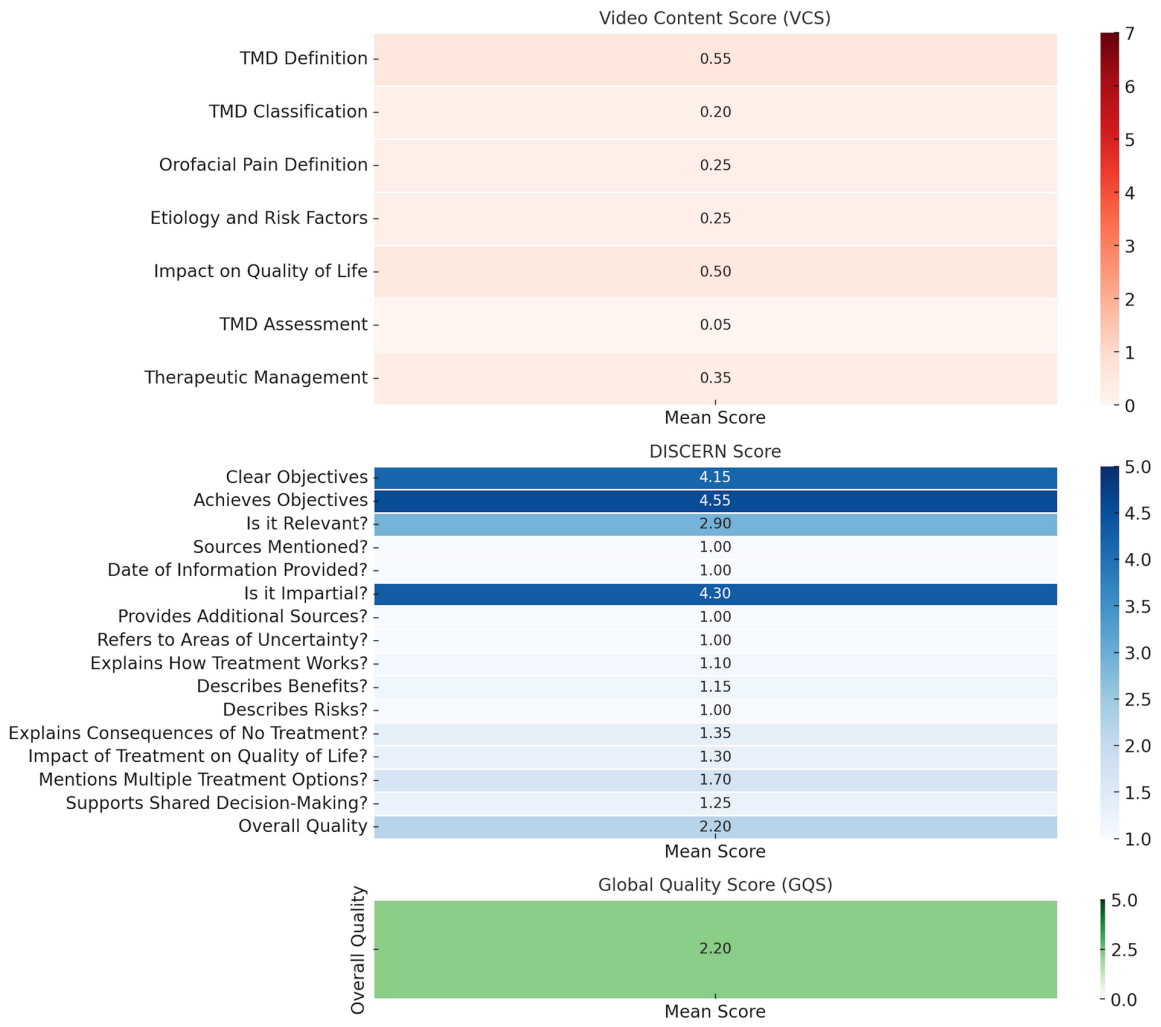
Heatmap illustrating the mean scores of TikTok videos on TMD, based on the Video Content Score (VCS), DISCERN Score, and Global Quality Score (GQS). The VCS highlights deficiencies in diagnostic assessment, classification, and discussion of etiology and risk factors. The DISCERN score reveals poor source citation, lack of date indication, and minimal acknowledgment of uncertainties and treatment risks, indicating low reliability of the information presented. The GQS demonstrates an overall low educational value of the videos.

Figure 3 illustrates the key deficiencies in the analyzed TikTok videos regarding TMD-related educational content.

3

VCS results reveal that the most critical gaps include the lack of proper assessment of TMD (0.05), inadequate classification of the disorder (0.20), and insufficient discussion of etiology and risk factors (0.25). These findings indicate that most videos fail to provide a comprehensive and structured explanation of TMD, particularly regarding diagnostic criteria and contributing factors. The DISCERN scores further highlight the low reliability of the information presented, with notably poor performance in critical domains such as source citation (1.00), date of information provided (1.00), acknowledgment of uncertainties (1.00), and explanation of treatment risks (1.10). These results suggest that most videos do not adhere to evidence-based guidelines and lack transparency regarding the credibility of the information. Finally, the GQS remains low (2.20), indicating that most videos provide limited educational value and do not meet high-quality standards for health communication.

**Figure 4:**
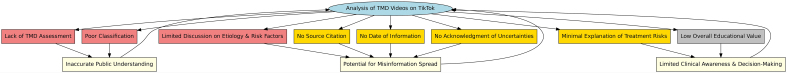
Flowchart illustrating the relationships between the main deficiencies identified in TikTok videos on TMD and their educational implications. The lack of structured content (VCS), unreliable information (DISCERN), and low overall quality (GQS) contribute to public misunderstanding, misinformation spread and limited clinical awareness. These findings emphasize the need for better content regulation and the dissemination of evidence-based educational materials on social media platforms.

Figure 4 illustrates the interconnections between the main deficiencies identified in the analyzed TikTok videos on TMD and their educational implications.

4

The VCS analysis highlights critical gaps in TMD assessment, classification, and discussion of etiology and risk factors, indicating a lack of structured and evidence-based content. Similarly, the DISCERN score reveals severe deficiencies in source citation, date of information, acknowledgment of uncertainties, and explanation of treatment risks, suggesting that most videos fail to provide reliable and transparent health information. Therefore, the GQS remains low, reflecting the overall limited educational value of the content. These deficiencies contribute to public misunderstanding of TMD, the spread of misinformation, and inadequate clinical awareness, reinforcing the need for better content regulation and the promotion of scientifically accurate educational materials on social media platforms.

## Discussion

This study evaluated the reliability and educational value of 98 TikTok videos on TMD, classified according to the content creator type: general users (4.1%), dental care professionals (60.2%), and other healthcare professionals (35.7%). The findings revealed that videos posted by other healthcare professionals received significantly more likes (p = 0.006) and had higher engagement (p = 0.004) compared to those created by dental care professionals. Overall, the median scores for all three evaluation criteria were low, indicating that most TikTok videos on TMD provide limited, unreliable, and poorly structured information. No statistically significant differences were observed in the educational quality scores (VCS: p = 0.453; DISCERN: p = 0.239; GQS: p = 0.341) among the groups of content creator type. Although videos created by healthcare professionals tended to present more structured and evidence-based content, the lack of significant differences between groups suggests a general deficiency in the quality of TMD-related content available on the platform. These results emphasize the need for greater oversight, content curation, and the promotion of scientifically accurate materials to ensure that social media users receive high-quality, evidence-based health information.

The predominance of healthcare professionals among content creators suggests a notable interest in using TikTok for TMD education, whereas the low representation of general users may indicate limited public awareness or engagement with the topic (33 - 35). Videos by other healthcare professionals garnered more likes and engagement than those by dental professionals, reinforcing previous findings that audience interaction is influenced more by presentation style than content accuracy. Interestingly, while general users' videos were often longer, they also received the highest number of views and likes, likely due to their more accessible language and patient-friendly approach. This contrasts with Fortuna et al. (33) where the longest videos found and analyzed were created by dental professionals and other health professionals, suggesting that health professionals may need more time to convey accurate and complete information.

Despite their expertise, dental professionals' videos often lacked engagement, possibly due to overly technical language, minimal visual aids, and a treatment-centered focus rather than comprehensive patient education (33). It is important to note that audience engagement, measured by likes and comments, may be more strongly influenced by presentation style than by the accuracy of the information provided (35). Nonetheless, a crucial point is that despite differences in engagement, there were no statistically significant differences in the educational quality of content among the creator groups. Additionally, popular videos were not necessarily the most reliable, emphasizing that engagement metrics alone are insufficient indicators of educational value (13 , 16 , 26 , 31).

The quality of the videos was assessed using tools such as DISCERN, the Global Quality Score (GQS), and other evaluation criteria, which frequently pointed to content that was low in quality, unreliable, and poorly structured. Despite possessing technical expertise, dental professionals often focus excessively on treatment, neglecting crucial information regarding etiology and risk factors (13 , 26). Furthermore, videos created by dentists tend to employ highly technical language and lack engaging visual resources, which results in lower audience engagement when compared to videos produced by other healthcare professionals or even laypersons (36). This highlights the need to reconsider their content production strategies, simplifying the language, using more engaging visuals, and providing more comprehensive information about dental care (16 , 35). It is also essential for health institutions and universities to take a more active role in creating high-quality content to improve the reliability and utility of online health information (13 , 37).

The overall educational quality of the analyzed videos was low, as indicated by a median GQS score of 2.00 (IQR = 1.00), classifying them within the "poor" to "moderate" range. This trend aligns with findings from other areas of dentistry, where educational quality remains suboptimal. For instance, more than half of the publications on botulinum toxin application for bruxism were classified as having poor or generally poor educational quality (GQS &lt; 3) (38). Similarly, studies on orthodontic treatments on YouTube reported GQS averages ranging from 2.6 to 3.229, while videos discussing pulpotomy and pulp capping had even lower mean GQS scores of 1.63 and 1.70, respectively (39 , 40). Despite the lack of statistically significant differences in quality scores between content creator groups (VCS: p = 0.453; DISCERN: p = 0.239; GQS: p = 0.341), videos produced by healthcare professionals showed a tendency toward higher quality, with more structured content aligned with scientific guidelines on TMD. In a bruxism and Instagram study, posts that incorporated personal experiences received higher GQS scores compared to those solely providing information or promotional content (38). A particularly concerning issue is the lack of distinction between bruxism and TMD, which can contribute to misunderstandings about these conditions and their management.

The dissemination of health-related information on TikTok has raised increasing concerns regarding its accuracy and reliability. The findings of this study indicate that most TMD-related videos on the platform exhibit low educational quality and poor reliability, as reflected in the low median scores of VCS, DISCERN, and GQS. These results align with previous research examining the credibility of medical content on TikTok, which has highlighted widespread misinformation and the lack of regulatory oversight (41 - 43).

A growing body of evidence suggests that misinformation and incomplete information are prevalent across various health topics on TikTok. Studies evaluating content on autism, diabetes, and varicoceles have found that a significant proportion of videos contain inaccurate or incomplete explanations, which may contribute to public misconceptions and misguided self-diagnosis (41 - 43). Similarly, analyses of myopia and sleep health content on the platform indicate that videos with higher engagement metrics do not necessarily provide scientifically accurate information, suggesting that TikTok's algorithm prioritizes entertainment and virality over accuracy (44 , 45). These findings reinforce concerns that TikTok's content ranking mechanisms may inadvertently amplify low-quality information, making it more difficult for the public to access credible and evidence-based health knowledge.

The misalignment between engagement and educational value observed in this study is consistent with prior research, demonstrating that popular content is not always synonymous with reliable health information. In many cases, videos created by non-health professionals gain widespread attention, while expert-driven content remains less visible and underutilized (46 , 47). This trend has also been observed in studies on cardiovascular disease, diabetes, and public health interventions, where layperson-generated videos often outnumber and outperform those created by medical professionals in terms of audience reach (46 - 48). Given this scenario, the potential consequences of misinformation are substantial, as individuals may adopt inaccurate health beliefs or engage in self-treatment strategies that lack scientific validation (48).

A crucial aspect of addressing this issue involves encouraging greater participation from healthcare professionals in content creation and dissemination. Research has shown that while expert-generated videos tend to have higher scientific rigor and alignment with medical guidelines, they often fail to capture audience engagement at the same level as non-expert content (44 , 46).

This highlights the need for improving the accessibility and appeal of professional health education videos, ensuring that credible information can effectively compete with viral but inaccurate content. Additionally, collaborations between healthcare professionals and influential content creators could serve as a viable strategy to bridge the gap between scientific accuracy and public engagement (46 , 47).

Furthermore, public perception of TikTok as a health information source warrants further investigation. Studies examining TikTok's influence on young women's health knowledge indicate that many users trust and act upon the information presented in videos without cross-referencing with medical sources, which may contribute to the spread of unverified health claims and self-diagnosis trends (48). This underscores the urgency of promoting digital health literacy initiatives to encourage critical evaluation of online health content (42 , 48). As TikTok continues to evolve as a dominant health information platform, ensuring the accuracy of medical content must be a priority for both researchers and policymakers.

Given these findings, efforts should focus on enhancing content regulation, promoting expert-reviewed health information, and increasing public awareness regarding misinformation risks. Healthcare organizations, universities, and public health institutions should consider implementing strategies to improve the visibility of credible content while developing educational campaigns to help users distinguish between reliable and misleading information (42 , 47).

Addressing these challenges is essential to improving the overall quality of health-related information on social media platforms and ensuring that evidence-based knowledge remains accessible to the public.

This study has some limitations that should be acknowledged. First, despite methodological efforts to create a neutral search environment, the TikTok algorithm remains an uncontrolled variable, potentially influencing the selection of videos based on engagement metrics rather than content accuracy. To reduce this, new accounts were created, and browsing history was cleared before conducting the searches. Second, although 98 videos were analyzed, the sample size may not fully capture the diversity of TMD-related content available on TikTok, especially in languages other than Portuguese and English. Third, the evaluation relied on validated scoring systems (VCS, DISCERN, and GQS), yet the inherent subjectivity of content assessment remains a potential limitation. To minimize bias, evaluators underwent calibration, and inter-rater reliability was ensured through high agreement levels (Cohen's kappa 0.86). Additionally, this study was restricted to TikTok, limiting generalizability to other social media platforms where different content dynamics may exist. Finally, while the study assessed the quality and reliability of the information, it did not explore how users interpret or apply this knowledge in clinical or self-care contexts. Future research should incorporate longitudinal designs and audience perception analyses to better understand the impact of social media health content on public awareness and patient decision-making.

## Conclusions

This study aimed to analyze the reliability and educational value of TikTok videos as a source of information on TMD. The findings indicate that most videos provide limited, unreliable, and poorly structured information, as reflected in the low median scores across the three evaluation criteria: Video Content Score (VCS), DISCERN, and Global Quality Score (GQS). While videos produced by healthcare professionals (dentists and other specialists) tended to exhibit higher content quality, there were no statistically significant differences between creator groups, reinforcing a general lack of well-validated and evidence-based content on the platform. Additionally, videos from other healthcare professionals showed higher engagement metrics than those from dental professionals, suggesting that audience interaction does not necessarily correlate with educational value. Given these results, TikTok cannot be considered a reliable source of educational information on TMD. The variability in content quality highlights the need for greater oversight, content validation, and initiatives to promote scientifically accurate health information on social media platforms. Encouraging the dissemination of expert-reviewed content and improving public awareness of misinformation are crucial steps toward enhancing the credibility and educational utility of digital health resources.

## Figures and Tables

**Table 1:** Comparison of Video Engagement Metrics, Content Popularity, and Visibility Across Different Categories of TikTok Content Creators.

	General Users (1)	Dental Care Professionals (2)	Other Health Care Professionals (3)	p-value (Kruskal-Wallis)
Number of followers	49400 (78982)	15000 (42526)	31200 (231700)	0.13
Duration in seconds	112 (92)	53 (42)	60 (26)	0.19
Upload Time	78 (176)	567 (426)	454 (404)	0.08
Number of views	16730 (30265)	18900 (51769)	47700 (174700)	0.11
Number of likes	478 (731)	263 (732)	862 (4277)	0.006*
Number o comments	46 (42)	18 (48)	35 (177)	0.16
Number of shares	38 (66)	26 (133)	103 (551)	0.07
Viewing Index	143.29 (269.13)	45.84 (152.04)	186.40 (673.41)	0.007*
Engagement Index	558.00 (842.75)	371.00 (858.00)	1164.00 (7007.00)	0.004*
Dunn 1 vs 2	-	-	-	0.68
Dunn 1 vs 3	-	-	-	0.29
Dunn 2 vs 3	-	-	-	0.002*

Note: Comparison of median (IQR) values for video characteristics across the three categories of content creators: General users, Dental care professionals, and other health care professionals. Statistical comparisons were performed using the Kruskal-Walli’s test, with significant differences indicated by p-values < 0.05. Post-hoc Dunn tests were conducted for the number of likes, revealing a significant difference between Dental care professionals and other health care professionals (p = 0.001).

## Data Availability

The datasets used and/or analyzed during the current study are available from the corresponding author.
